# Geographical variations in risk factors associated with HIV infection among drug users in a prefecture in Southwest China

**DOI:** 10.1186/s40249-015-0073-x

**Published:** 2015-09-02

**Authors:** Yi-Biao Zhou, Qi-Xing Wang, Song Liang, Yu-Han Gong, Mei-Xiao Yang, Yue Chen, Shi-Jiao Nie, Lei Nan, Ai-Hui Yang, Qiang Liao, Yang Yang, Xiu-Xia Song, Qing-Wu Jiang

**Affiliations:** Department of Epidemiology, School of Public Health, Fudan University, 138 Yi Xue Yuan Road, Shanghai, 200032 China; Key Laboratory of Public Health Safety, Fudan University, Ministry of Education, Shanghai, China; Center for Tropical Disease Research, Fudan University, Shanghai, China; Center for Disease Prevention and Control of the Liangshan Yi Autonomous Prefecture, Sichuan, China; Department of Environmental and Global Health, College of Public Health and Health Professions, University of Florida, Gainesville, FL USA; Emerging Pathogens Institute, University of Florida, Gainesville, FL USA; Xuhui Center for Disease Prevention and Control, Shanghai, China; School of Epidemiology, Public Health and Preventive Medicine, Faculty of Medicine, University of Ottawa, Ottawa, ON Canada; Department of Biostatistics, University of Florida, Gainesville, FL USA

**Keywords:** Human immunodeficiency virus, Drug users, Geographically weighted logistic regression, Geographical variation, Ethnic epidemiology

## Abstract

**Background:**

Previous studies have shown inconsistent or even contradictory results for some risk factors associated with HIV infection among drug users, and these may be partially explained by geographical variations.

**Methods:**

Data were collected from 11 methadone clinics in the Liangshan Yi Autonomous Prefecture from 2004 to 2012. A non-spatial logistical regression model and a geographically weighted logistic regression model were fitted to analyze the association between HIV infection and specific factors at the individual level.

**Results:**

This study enrolled 6,458 patients. The prevalence of HIV infection was 25.1 %. The non-spatial model indicated that being divorced was positively associated with HIV infection. The spatial model also showed that being divorced was positively associated with HIV infection, but only for 49.4 % of individuals residing in some northern counties. The non-spatial model suggested that service sector work was negatively associated with HIV infection. However, the spatial model indicated that service work was associated with HIV infection, but only for 23.0 % of patients living in some western counties. The non-spatial model did not show that being married was associated with HIV infection in our study field, but the spatial model indicated that being married was negatively associated with HIV infection for 12.0 % of individuals living in some western counties. For other factors, the non-spatial and spatial models showed similar results.

**Conclusion:**

The spatial model may be useful for improving understanding of geographical heterogeneity in the relationship between HIV infection and individual factors. Spatial heterogeneity may be useful for tailoring intervention strategies for local regions, which can consequently result in a more efficient allocation of limited resources toward the control of HIV transmission.

**Electronic supplementary material:**

The online version of this article (doi:10.1186/s40249-015-0073-x) contains supplementary material, which is available to authorized users.

## Multilingual abstracts

Please see Additional file 1 for translations of the abstract into the six official working languages of the United Nations.

## Background

In recent decades, illicit drug abuse has become a serious social and public health problem worldwide. It directly cost the world 20.0 million disability-adjusted life years (DALYs) and accounted for 0.8 % of all-cause DALYs in 2010 [1]. There are about 2.4 million injecting drug users in China [2].

Human immunodeficiency virus (HIV), the causative pathogen of acquired immune deficiency syndrome (AIDS), is associated with intravenous drug use (IDU) and sharing of syringes. Approximately 34 million people are living with HIV worldwide [3]. In China, there were an estimated 780,000 individuals living with HIV, including 154,000 with AIDS in 2011, with 28.4 % of these infected from IDU [4]. By the end of January 2015, there were 508,864 reported existing live HIV infected patients, including 206,366 with AIDS, with 160,288 patients dying of AIDS in China [5]. The prevalence of HIV infection varies geographically [4, 6–8]. Study results showing associations of HIV infection with demographic, socioeconomic, and drug-related behavioral factors have not always been consistent across regions [7, 9–11], and sometimes were contradictory, which might have resulted from geographical heterogeneity. There are significant global and local spatial autocorrelations for HIV infection [4, 12]. Spatial autocorrelations could cause biases for association estimates and their standard errors if they are not properly accounted for [13, 14]. In the current study, we used a geographically weighted logistic regression (GWLR) model to determine the associations between demographic, socioeconomic, and drug use behavioral characteristics and HIV infection among drug users in the Liangshan Yi Autonomous Prefecture in Southwest China, taking geographical variations into consideration. The Yi people, with a population of approximately eight million, are the seventh largest ethnic group of the 55 ethnic minority groups in China. They live primarily in rural areas (usually in mountainous regions) of Sichuan, Yunnan, Guizhou, and Guangxi provinces. For the Yi ethnic group, rigid rules are stipulated for marriages within the same rank. The current analysis is important for developing more effective HIV prevention programs that are tailored to the needs of diverse groups and consequently increasing access to health services for those who need it most.

## Methods

### Study area and population

**Fig. 1:**
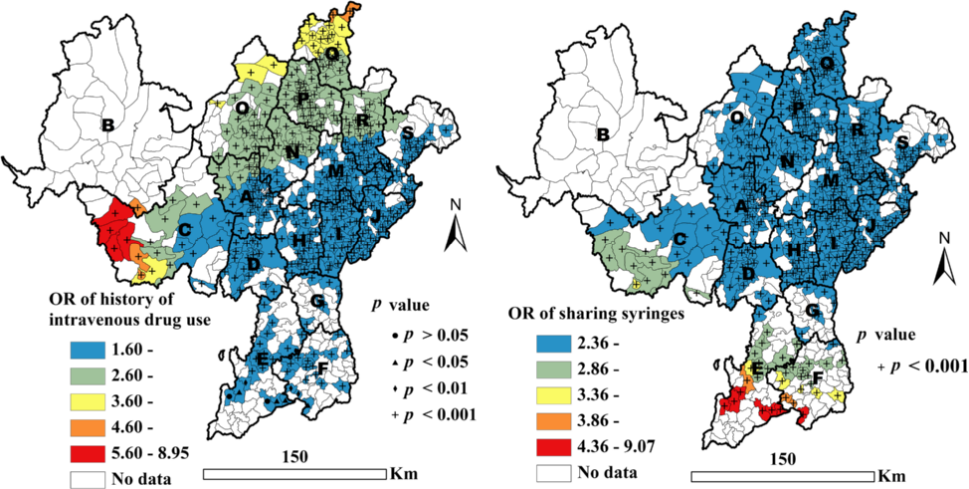
Geographical distribution of adjusted ORs showing history of IDU and sharing of syringes, as associated with HIV infection

The Liangshan Yi Autonomous Prefecture is home to one of the largest illicit drug production and distribution centers in China. It lies on the border of Sichuan and Yunnan provinces in Southwest China, where HIV/AIDS is prevalent. It has a population of 4.9 million people and the largest population of ethnic Yi in China. This prefecture consists of 618 townships, 16 counties (B-J, M-S counties), and one city (A city) (Fig. 1). Five of the counties have more than 75 % of ethnic Yi, who are mostly located in the northeastern part of the prefecture and have the highest risk of HIV infection [4, 13]. In order to control the dual epidemics of HIV/AIDS and drug use, a methadone clinic was first established in the prefecture in 2004. As of late 2012, there were 10 fixed national methadone clinics and one mobile national methadone clinic in the prefecture. All patients who attended these 11 clinics from 2004 to 2012 were included in the present study and were examined for HIV infection, i.e., all patients were encouraged to have a test for HIV one month after the first visit to the methadone clinics. First, HIV antibody was tested for, then, positive test results were further confirmed using the Western blot analysis. All participants were also asked to take part in a questionnaire survey. The questionnaires were administered face-to-face in a private room by a same-sex interviewer trained in how to administer questionnaires. Information collected included data on demographic and socioeconomic factors, drug use behavioral factors, residency of drug users, etc. The participants’ residency information was taken from their identification cards. Detailed information about the infrastructure and procedures of these clinics, and how they related to the participants has been provided elsewhere [4, 13, 15–17]. Ethical approval for methadone maintenance treatment was provided by the institutional review board of the National Center for AIDS/STD Control and Prevention, China Center for Disease Control. Each participant gave his/her written informed consent.

### Data collection

The current study was approved by the Center for Disease Prevention and Control of the Liangshan Yi Autonomous Prefecture. Data were extracted from the national methadone database, and included information on HIV infection, demographic and socioeconomic factors, drug use behavioral factors, and residency of all drug users who were treated at the 11 clinics between 2004 and 2012 [4, 15]. A digitized polygon map of the prefecture at a scale of 1:250,000 was obtained from the Shanghai Digital Bitmap Information Science Co., Ltd; the township is used as the basic geographical unit in this analysis. The latitude and longitude of the center of each township were extracted from this map. The location of each individual was defined as the geographical coordinates (i.e., latitude/longitude) of the center of the township in which he/she lived. All data were entered into Microsoft Excel version 2010 (Microsoft Corp., Redmond, WA, USA).

### Analytic strategy

A GWLR model was used to examine the associations between HIV infection and individual factors. The GWLR model is expressed as follows [18]:

$$ \log \left(\frac{P\left({y}_i=1\right)}{1-P\left({y}_i=1\right)}\right)=\left.{\beta}_{0i}\left({u}_i,{v}_i\right)+{\displaystyle {\sum}_{j=1}^k{\beta}_{ij}\left({u}_i,{v}_i\right){x}_{ij}}\right) $$

Here, *y*_*i*_, *x*_*ij*_, (*u*_*i*_*,v*_*i*_), and *β*_*ij*_ signify the HIV infection for each individual i, a set of independent variables (j = 1, …, k) for individual i, the x-y coordinates of individual i, and the estimated effect of independent variable j for individual i, respectively.

Current explanatory variables grasped three domains of interest. The first domain was related to drug use behaviors that embody IDU, sharing of syringes, and drug rehabilitation. The second domain was related to demographic characteristics, including age (<30, 30–39, 40–49, and ≥50 years), gender (male, female), ethnicity (Yi, Han, other), and marital status (single, married, divorced, widowed). Three dummy variables were created for age before entering into the models; they were 30–39 (1 = yes, 0 = no), 40–49 (1 = yes, 0 = no), and ≥50 years (1 = yes, 0 = no) respectively, and the <30 years group served as the reference group. Gender was a dummy variable with female used as the reference group. Ethnicity was categorized into Yi, Han (reference group), and others using two dummy variables. Marital status was classified as single (reference), married, divorced, and widowed using three dummy variables. The third domain was related to socioeconomic factors such as occupation and education. Occupation was categorized as farmer, service sector worker, unemployed, and others (reference), with this category including factory worker, government employee, etc. (three dummy variables). Education status was categorized into four sub-categories (three dummy variables): no schooling (reference), elementary school, junior high school, and senior high school or above.

Non-spatial logistical regression analysis was carried out using the SPSS Statistical Package for Social Sciences (SPSS Inc., Chicago, IL, USA, 2007). The GWLR model was used with the iteratively reweighted least squares method in GWR 4.0 software (https://geodacenter.asu.edu/software/downloads/gwr_downloads). The kernel type and function for geographical weighting to estimate local (i.e., in a space rather than the space as a whole) coefficients and their bandwidth size was adaptive bisquare. The bandwidth was the number of nearest neighbors being included in the bisquare kernel. The golden section search method was used to automatically determine the best bandwidth size based on the Akaike information criterion (AICc) (small sample-bias-corrected AIC), with the bandwidth with the lowest AICc used in the analysis. A distance-based weighting scheme was used to assign weights in the GWLR model with the bisquare kernel and bandwidth. Geographical variability for each varying coefficient was tested by model comparison [19]. The coefficient of an explanatory variable in the GWLR model was the change in the log odds of the response given a unit change in that variable. Then, the exponentiation of the coefficient can be used to find the odds ratio (OR) corresponding to a unit change in the variable.

Variables were selected as follows: First, all variables were included in the GWLR model. Then, the variable with the largest *p*-value was removed from the model until, and only, the variables with some of the local *p-*values less than 0.1 remained. Based on the geographical coordinates (i.e., latitude/longitude) of each individual, the local mean estimates (OR) and *p*-values for each individual were visualized on a map to demonstrate the geographical variations in the associations between HIV infection and individual factors using ArcGIS 10.0 software (Environmental Systems Research Institute, Inc., Redlands, CA, US). The GWLR model is relatively insensitive to the choice of kernel function (e.g., bisquare versus Gaussian) [18].

## Results

### Prevalence of HIV infection

Of the 6,458 drug users, 45.9 % had a history of IDU and 18.1 % shared syringes. The prevalence of HIV infection was 25.1 % (1,622/6,458), and was higher in males (26.4 %) than in females (17.2 %) (*χ*^*2*^ = 36.2, *p* < 0.001). The prevalence decreased with increasing age (*χ*_*trend*_^*2*^ = 29.3, *p* < 0.001) and increasing education level (*χ*_*trend*_^*2*^ = 296.8, *p* < 0.001). The prevalence was higher in Yi people (28.4 %) than other ethnicities (*χ*^*2*^ = 184.7, *p* < 0.001), and in farmers than in non-farmers (see Table 1).

**Table 1:** The characteristics of the national methadone clients in Southwest China

Study variable	Number of clients examined	Infection prevalence (%)	*χ* ^2^	P
Drug use behaviors				
History of intravenous drug use				
Yes	2963	35.7	328.6	<0.001
No	3491	16.1		
History of sharing syringes				
Yes	1166	49.1	433.6	<0.001
No	5292	19.8		
History of drug rehabilitation				
Yes	2891	25.4	0.2	0.649
No	3567	24.9		
Demographic factors				
Gender				
Males	5537	26.4	36.2	<0.001
Females	921	17.2		
Age				
< 30	2877	27.5	34.3	<0.001
30-39	2754	24.6		
40-49	738	20.2		
≥ 50	89	6.7		
Marital status				
Single	1297	22.4	10.4	0.016
Married	4889	25.7		
Divorced	204	23.5		
Widowed	68	35.3		
Ethnicity				
Yi	5355	28.4	184.7	<0.001
Han	1055	8.6		
Other	48	18.8		
Socioeconomic factors				
Employment				
Farming,	5151	28.5	161.2	<0.001
Service-sector,	194	7.2		
Unemployed	858	12.8		
Other	255	11.0		
Educational attainment				
No schooling	1586	37.3	308.1	<0.001
Primary school	2885	27.2		
Junior high school	1402	13.4		
Senior high school or above	585	9.7		

### Non-spatial logistical regression

Table 2 shows the factors that were significantly associated with HIV infection. Histories of IDU and syringe sharing were positively associated with HIV infection (*p* < 0.001). Being divorced or widowed was associated with a greater risk of HIV infection when compared with being single (*p* < 0.01). It was not so for being married (*p* > 0.05). Male drug users or ethnic Yi individuals had a greater risk of HIV infection than female drug users (*p* < 0.01) or ethnic Han individuals (*p* < 0.001). Service sector work and some school education were negatively associated with HIV infection (*p* < 0.001) (see Table 2).

**Table 2:** Non-spatial logistical regression results of HIV infection

Study variable	B	Wald *x* ^*2*^	*P* value	Adjusted	Adjusted
				OR	OR 95%CI
Constant	−2.86	319.254	<0.001	0.06	–
Drug use behaviors					
History of intravenous drug use (1 = yes, 0 = no)	0.96	180.220	<0.001	2.61	2.27–3.00
History of sharing syringes (1 = yes, 0 = no)	0.96	135.671	<0.001	2.60	2.22–3.06
Demographic factors					
Marital status (ref = single)					
Divorced (1 = yes, 0 = no)	0.51	7.116	0.008	1.66	1.14–2.41
Widowed (1 = yes, 0 = no)	0.76	7.213	0.007	2.14	1.23–3.72
Gender (1 = males, 0 = females)	0.41	16.799	<0.001	1.51	1.24–1.84
Ethnicity (ref = Han)					
Yi (1 = yes, 0 = no)	1.32	115.348	<0.001	3.76	2.95–4.79
Socioeconomic factors					
Employment (ref = other, such as factory worker, government employee, etc.)					
Service sector (1 = yes, 0 = no)	−0.94	10.271	<0.001	0.39	0.22–0.70
Educational attainment (ref = no school)					
Primary school (1 = yes, 0 = no)	−0.36	23.801	<0.001	0.70	0.61–0.81
Junior high school (1 = yes, 0 = no)	−1.12	114.524	<0.001	0.33	0.27–0.40
Senior high school or above (1 = yes, 0 = no)	−1.43	80.983	<0.001	0.24	0.18–0.33

### Multivariate spatial logistical regression

In the present study, the optimal bandwidth size was 170 (persons). The value of AICc in the global regression was 6236.7 compared with 6218.1 in the local regression, indicating that the fitness of the local regression was better than the global regression. Table 3 shows the factors that were significantly and locally associated with HIV infection. Sharing syringes was found to be positively associated with HIV infection for all individuals, with adjusted OR values ranging from 2.36 to 9.07 (see Table 3 and Fig. 1). It was also found that a history of IDU was positively associated with HIV infection for 99.9 % of drug users. The remaining 0.1 % all resided in E county (see Table 3 and Fig. 1). For 99.9 % of the individuals in this study, drug rehabilitation was not found to be significantly associated with HIV infection (*p* > 0.05) (see Table 3).

**Table 3:** Results of the local estimates from the GWLR model of HIV infection

Study variable	Minimum		Medium		Maximum		Significant proportion
	B	OR	B	OR	B	OR	(%)
Constant	−4.59		−3.00	0.05	−2.46	-	100.0
Drug use behaviors							
History of intravenous drug use (1 = yes, 0 = no)	0.47	1.60	0.87	2.39	2.19	8.94	99.9
History of sharing syringes (1 = yes, 0 = no)	0.86	2.36	0.89	2.44	2.21	9.07	100.0
History of drug rehabilitation (1 = yes, 0 = no)	−0.09	0.91	0.09	1.09	1.18	3.26	0.1
Demographic factors							
Marital status (ref = single)							
Married (1 = yes, 0 = no)	−0.48	0.62	−0.09	0.91	0.38	1.46	12.0
Divorced (1 = yes, 0 = no)	0.00	1.00	0.43	1.54	0.72	2.05	49.4
widowed (1 = yes, 0 = no)	−0.16	0.86	0.73	2.08	1.62	5.04	61.1
Gender (1 = males, 0 = females)	0.07	1.07	0.46	1.58	0.52	1.68	98.1
Ethnicity (ref = Han)							
Yi (1 = yes, 0 = no)	1.04	2.83	1.29	3.63	2.03	7.61	100.0
Socioeconomic factors							
Employment (ref = other, such as factory worker, government employee, etc.)							
Service sector (1 = yes, 0 = no)	−1.44	0.24	−0.68	0.51	0.81	2.25	23.0
Educational attainment (ref = no school)							
Primary school (1 = yes, 0 = no)	−0.88	0.41	−0.33	0.72	0.26	0.76	99.9
Junior high school (1 = yes, 0 = no)	−1.31	0.27	−1.08	0.34	−0.83	0.44	100.0
Senior high school or above (1 = yes, 0 = no)	−1.52	0.22	−1.35	0.26	−0.85	0.43	99.8

Note: Minimum, Medium and Maximum in the table are the minimum local estimate value, the median local estimate value, and maximum local estimate value respectively; OR in the table is adjusted OR; Significant proportion in the table indicates the proportion of individuals whose factor is associated significantly with HIV infection

**Fig. 2:**
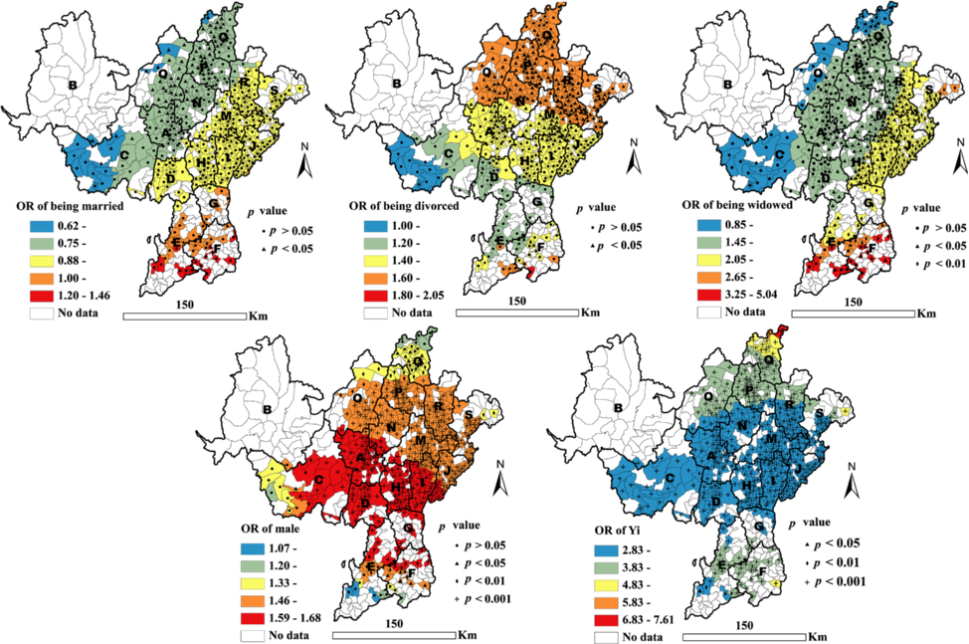
Geographical distribution of adjusted ORs of marital status, male gender, and Yi ethnicity, as associated with HIV infection

In terms of the demographic factors, being married was found to be negatively associated with HIV infection for 12 % of all individuals living in western counties (C, O, P, and Q counties) and A city (*p* < 0.05) (see Table 3 and Fig. 2). Being divorced was found to be positively associated with HIV infection for 49.4 % of all patients living in northern counties (*p* < 0.05) (see Table 3 and Fig. 2). Being widowed was also found to be positively associated with HIV infection for 61.1 % of all patients residing in eastern counties (*p* < 0.05) (see Table 2 and Fig. 2). Male participants had a significantly higher risk of HIV infection (*p* < 0.05) (see Table 3 and Fig. 2). Ethnic Yi participants were more likely to be infected with HIV than ethnic Han participants (*p* < 0.05) (see Table 3 and Fig. 2).

**Fig. 3:**
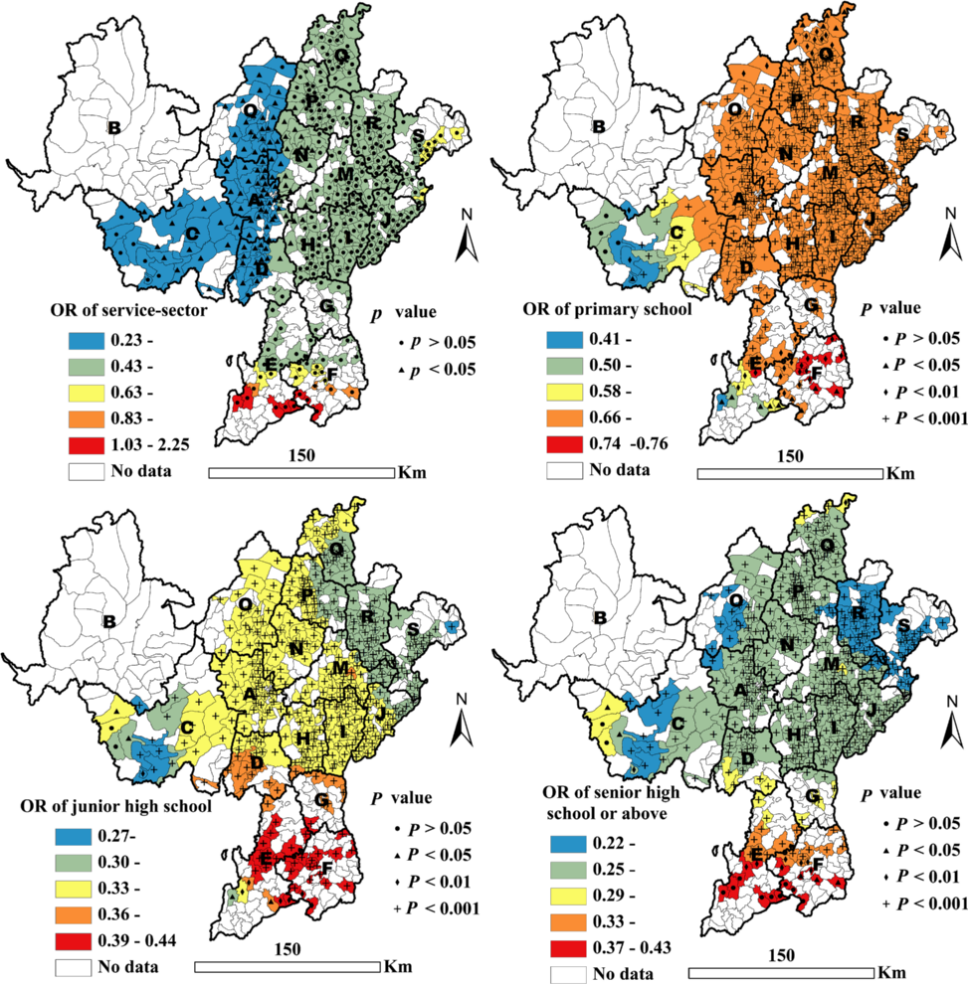
Geographical distribution of adjusted ORs of being employed in the service sector and level of educational, as associated with HIV infection

In terms of the socioeconomic factors, service sector work was found to be negatively associated with HIV infection for 23.0 % of all drug users living in western counties compared to other occupations such as factory workers or government workers (*p* < 0.05) (see Table 3 and Fig. 3). Compared with no schooling, having primary school education or above was negatively associated with HIV infection for most of the population (*p* < 0.05); OR values decreased with an increasing level of education (see Table 2 and Fig. 3).

## Discussion

The GWLR model showed geographical variations in the risk factors associated with HIV infection, something that was not indicated by the non-spatial logistic regression analysis. For example, the non-spatial analysis indicated that being divorced or widowed was associated with HIV infection, as a previous study also showed [20]. However, the GWLR model showed that being divorced was positively associated with HIV infection for only 49.4 % and being widowed was associated with HIV infection for only 61.1 % of all drug users in this study, respectively. Geographical variations might explain, at least in part, the inconsistent results for the associations between demographic factors and HIV infection, as also shown in previous studies [7, 9, 11]. The geographical variations in the association estimates in the current study might be related to different transmission patterns of HIV infection in the study area [4, 13]. For example, heterosexual intercourse was found to be a major route of transmission in some eastern counties (e.g., I, M) and IDU was a dominant route of transmission in some northwestern counties (e.g., A city) [4, 15]. The first case of HIV was found in the eastern M county in 1995, and some older AIDS patients had begun to die in some of the eastern counties in recent years (e.g., I and M). These might be plausible explanations for the association between being widowed and HIV infection in some eastern counties. However, this speculation needs confirmation.

For drug use behaviors, the GWLR and non-spatial logistic regression models showed similar results regarding injection history and sharing of syringes as global factors associated with HIV infection, which is in accordance with results from previous studies [7, 13]. Consistent results were also obtained for ethnicity. A previous ecological study showed that the relative number of ethnic Yi individuals among drug users was closely related to the prevalence of HIV infection in this prefecture [13]. Another study also showed that 82.9 % of the drug users in the prefecture were of Yi ethnicity, and the areas with the highest prevalence of HIV infection were mainly distributed in the I, M, R, and P counties, where the proportion of Yi ethnic individuals was over 50 % [4]. These findings all indicate that the Yi ethnic group is a high-risk population for HIV infection. The Liangshan Yi Autonomous Prefecture has experienced an alarming increase in HIV infection since the first HIV case was found in M county, where 97.1 % were Yi people in 1995. By the end of 2011, the cumulative number of cases with positive HIV infection was over 25,000 [4], with most of these in the M, R, and I counties, which has a vast majority of the Yi population.

Poverty, culture, and tradition might explain why the Yi people have a high risk of HIV infection. Previous studies have shown that poverty is associated with HIV infection, and some dimensions of being poor have been found to increase the risk and vulnerability to HIV [21–24]. Most Yi ethnic individuals reside in the poorest rural and minority areas (e.g., M, I, H, and R counties, which are all beneath the national poverty line). Arranged marriage between individuals of the same social status is common in the Yi community, and marriage within the social stratum may reinforce barriers to advancement and education. There is a custom of marrying the widow of one’s brother to maintain his line. For example, a man may marry his brother’s widow even if his brother, his sister-in-law, or both are known to have been HIV positive. There is also some acceptance of casual sex and condom use is infrequent [25].

Both the GWLR and non-spatial logistic regression models indicated that the odds of HIV infection were higher in male than female drug users, and the association tended to be stronger than previously estimated [7]. A previous township-level ecological study showed that a proportion of male drug users were positively associated with the prevalence of HIV infection in northwestern counties, but the opposite trend was observed in eastern counties [13]. Another study showed that the percentage of existing HIV infections that could be attributed to sexual transmission was 88 % in both I and M counties, 55 % in P county, 67 % in R county, and only 38 % in A city [4]. It is likely that there were different patterns of transmission of HIV infection in this area. Increasing the level of education might be helpful in preventing HIV infection in the Liangshan Autonomous Yi Prefecture, which is in accordance with some existing reports [21, 23, 26, 27].

Our findings may have some important implications: 1) Spatial autocorrelation and heterogeneity might exist for demographic and socioeconomic factors associated with HIV infection, which should not be ignored. 2) The role of geographical variations in factors might provide some guidance for tailoring site-specific intervention strategies to better prevent or control HIV transmission in areas with limited resources. For example, drug users who are widowed and reside in eastern counties, or who are divorced and live in northern counties should be prioritized for intervention. 3) It is important to increase the level of education for people living in the Liangshan Yi Autonomous Prefecture. Most ethnic Yi individuals in the prefecture are either illiterate or have acquired only primary school education.

The present study had several limitations. First, information regarding sexual behavior, which can include important risk factors for HIV transmission, was not available. The effects of sexual risk factors on HIV infection could not be estimated or controlled, however, homosexuality (especially gay men) was rarely found in the study population. Second, not all the drug users in the region were enrolled in the national methadone clinics, and we are not able to confirm that our study population is a representative sample of community-dwelling drug users. However, one study did report that the prevalence of HIV among patients at the methadone clinics was not significantly different from that among other drug users [28]. Third, the study took place over a long time period, and misclassification bias might occur due to the change in diagnosis tests of HIV, however, this misclassification might be a nonspecific misclassification that made ORs to be close to 1. Fourth, the GWLR model might have some limitations including risk of multicollinearity among local estimates [18].

## Conclusion

Studying geographical variations in risk factors associated with HIV infection might provide some guidance for tailoring site-specific intervention strategies to better control and prevent HIV transmission in areas with limited resources. Spatial autocorrelation and heterogeneity are important factors to consider in future studies of risk factors for HIV infection.

## Additional file

Additional file 1:
**Multilingual abstracts in the six official working languages of the United Nations.** (PDF 312 kb)

## Abbreviations

AICc: Akaike information criterion
AIDS: Acquired immune deficiency syndrome
DALY: Disability-adjusted life year
GWLR: Geographically weighted logistic regression
HIV: Human immunodeficiency virus
IDU: Intravenous drug use
OR: Odds ratio
