# Supplementary material for: Geographical variations in risk factors associated with HIV infection among drug users in a prefecture in Southwest China
Source: Infect Dis Poverty. 2015 Sep 2;4:38. doi: 10.1186/s40249-015-0073-x (PMC4557839; doi:10.1186/s40249-015-0073-x)
Supplement: Additional file 1: — Multilingual abstracts in the six official working languages of the United Nations. (PDF 312 kb) [file 40249_2015_73_MOESM1_ESM.pdf]

Translation of the abstract into the six official working languages of the United Nations

الإختلافات الجغرافية في عوامل الخطر المرتبطة بفيروس نقص المناعة البشرية بين متعاطي المخدرات في محافظة فى جنوب غرب الصين

يي بياو تشو، تشى شينغ وانغ، سونغ يانغ، يو هان غونغ، مي شياو يانغ، يوي تشين، شى جياو نيه، لى نان، أى هوي يانغ، تشيانغ لياو، يانغ يانغ، شيوى شيا سونغ و تشينغ وو جيانغ

ملخص

الخلفية: أظهرت الدراسات السابقة نتائجا متضاربة أو متناقضة لبعض عوامل الخطر المرتبطة بالعدوى بفيروس نقص المناعة بين متعاطي المخدرات والتي يمكن تفسيرها جزئيا بالإختلافات الجغرافية.

الأساليب: تم جمع البيانات من 11 عيادة ميثادون بناحية يانغشان يي ذاتية الحكم في الفترة من 2004 إلى 2012وتم تجهيز نموذج الإنحدار اللوجستي الغير مكانى ونموذج الانحدار اللوجستي المرجح جغرافيا لتحليل العلاقة بين الإصابة بفيروس نقص المناعة البشرية وبعض العوامل المحددة على مستوى الأفراد .

النتائج: عدد المرضى المسجلين فى هذه الدراسة كان 6458 مريضا وكان معدل انتشار العدوى بفيروس نقص المناعة البشرية بينهم 25.1٪، أظهر النموذج غير المكاني أن فيروس نقص المناعة البشرية يرتبط إيجابيا مع حالات الطلاق، كما أظهر النموذج المكاني أيضا إرتباط فيروس نقص المناعة البشرية إيجابيا مع حالات الطلاق ولكن فقط فى نسبة 49.4٪ من الأفراد الذين يقيمون في بعض المقاطعات الشمالية، واقترح النموذج غير المكاني إرتباط عدوى فيروس نقص المناعة البشرية سلبيا بقطاع الأعمال الخدمية بينما أشار النموذج المكاني إلى إرتباط هذا القطاع بعدوى فيروس نقص المناعة البشرية فى 23.0٪ من المرضى الذين يعيشون في بعض المقاطعات الغربية، لم يُظهر النموذج غير المكاني إرتباط عدوى فيروس نقص المناعة البشرية بالزواج في مجال دراستنا، بينما أظهر النموذج المكاني أن عدوى فيروس نقص المناعة البشرية يرتبط سلبيا مع الزواج فى نسبة 12.0٪ من الأفراد الذين يعيشون في بعض المقاطعات الغربية، أظهرت النماذج غير المكانية والمكانية نتائجا مماثلة فى بعض العوامل الأخرى.

الاستنتاج: النموذج المكاني قد يكون مفيدا في تحسين فهم التباين الجغرافي في العلاقة بين الإصابة بفيروس نقص المناعة البشرية والعوامل الفردية كما أن التباين المكاني قد يكون مفيدا لتصميم استراتيجيات التدخل في المناطق المحلية، وبالتالي يؤدي إلى زيادة كفاءة تخصيص الموارد المحدودة نحو مكافحة فيروس نقص المناعة البشرية.

Translated from English version into Arabic by Mohamed R. Habib, through

CDC CHINESE CENTER FOR DISEASE CONTROL AND PREVENTION
NATIONAL INSTITUTE OF PARASITIC DISEASES

中国西南部某自治州吸毒人群 HIV 感染危险因素的地理分布变化

周艺彪，王启兴，梁松，龚煜汉，杨美霞，陈跃，聂世娇，南磊，杨爱辉，廖强，杨洋，宋秀霞，姜庆五

摘要

背景：一些研究显示，有关吸毒者 HIV 感染的一些危险因素的研究结果不一致，甚至互相冲突，这也许部分跟研究现场地理位置的变化有关。

方法：收集 2004－2012 年凉山州 11 个美沙酮门诊的数据，使用非空间的 logistic 回归模型和地理加权的 logistic 回归模型在个体水平上分析吸毒人群的 HIV 感染的危险因素。

结果：本次研究共分析了6458名美沙酮门诊病人，HIV感染率为25.1%。非空间回归模型显示，离婚是HIV感染的危险因素，但空间回归模型显示，离婚仅跟49.4%居住在一些北部县的美沙酮门诊病人HIV感染有关。非空间回归模型结果也显示，从事服务业与HIV感染阴性相关，但空间回归模型结果显示，从事服务业仅跟23.0%居住在一些西部县的美沙酮门诊病人感染HIV有关。在我们的研究现场，非空间模型没有发现结婚

与HIV感染有关，但空间模型结果显示，对12.0%居住在一些西部县的美沙酮门诊病人，结婚是HIV感染的保护因素。对其它因素，非空间回归模型与空间回归模型的结果相似。

结论: 空间回归模型有助于理解HIV感染危险因素在地理分布上的异质性，这种危险因素的地理异质性也许有利于当地政府因地制宜地制定艾滋病的防治策略，更合理地安排使用有限的卫生资源去控制HIV的传播。

Translated from English version into Chinese by Yi-Biao Zhou, through

Variations géographiques des facteurs de risque associés à l'infection par le VIH chez des usagers de drogue d'une préfecture du Xinan

Yi-Biao Zhou, Qi-Xing Wang, Song Liang, Yu-Han Gong, Mei-Xiao Yang, Yue Chen, Shi-Jiao Nie, Lei Nan, Ai-Hui Yang, Qiang Liao, Yang Yang, Xiu-Xia Song, Qing-Wu Jiang

Résumé

Contexte : Les résultats inconsistants, voire même contradictoires, obtenus par de précédentes études quant à certains facteurs de risque associés à l'infection par le VIH chez des usagers de drogue pourraient être partiellement expliqués par des variations géographiques.

Méthodes : Les données ont été recueillies auprès de onze centres de distribution de méthadone dans la préfecture autonome yi du Liangshan entre 2004 et 2012. Un modèle de régression logistique non spatiale et un modèle de régression logistique géographiquement pondérée ont été mis en place pour analyser le lien entre l'infection par le VIH et les facteurs spécifiques au niveau individuel.

Résultats : Cette étude a compté 6 458 patients. La prévalence de l'infection par le VIH était de 25,1 %. Selon le modèle non spatial, le fait d'être divorcé était positivement associé à l'infection par le VIH. Le modèle spatial a également démontré que le fait d'être divorcé était positivement lié à l'infection par le VIH, mais seulement pour 49,4 % des individus résidant dans certains comtés du nord. Le modèle non spatial a suggéré que travailler dans le secteur tertiaire était négativement associé à l'infection par le VIH. Cependant, le modèle spatial a également démontré qu'un emploi tertiaire était positivement lié à l'infection par le VIH, mais seulement pour 23,0 % des individus résidant dans certains comtés de l'ouest. Le modèle non spatial n'a pas indiqué d'association entre le fait d'être marié et l'infection par le VIH dans notre champ d'étude, tandis que le modèle spatial a indiqué une association négative entre le fait d'être marié et l'infection par le VIH pour 12,0 % des individus vivant dans certains comtés de l'ouest. Les deux modèles, non spatial comme non spatial, ont obtenu des résultats similaires quant aux autres facteurs.

Conclusion : Le modèle spatial pourrait permettre une meilleure compréhension de la diversité géographique de la relation entre l'infection par le VIH et les facteurs individuels. La diversité spatiale pourrait s'avérer utile pour élaborer des stratégies d'intervention sur mesure selon les régions, ce qui peut ensuite résulter en une allocation plus efficace des ressources visant à contrôler la transmission du VIH.

Translated from English version into French by Clémentine Choubrac, through

Географическая изменчивость в факторах риска, ассоциируемых с ВИЧ-инфекцией, среди наркоманов в Юго-западном Китае

Ий Бяо Чжоу, Ци-Син Ван, Сун Лянь, Ю-Хань Гун, Мей-Сяо Янь, Ю Чен, Ши-Цзяо Ни, Лей Нан, Ай-Хуэй Янь, Цзян Лао, Янь Янь, Сю-Ся Сун, Цин-Ву Цзян

Резюме

Базовая проблематика: Предыдущие исследования показали непоследовательные, и даже противоречивые, результаты по некоторым факторам риска, ассоциируемым с ВИЧ-инфекцией, среди наркоманов. Такие результаты частично могут быть отнесены за счет географической изменчивости.

Методы: Данные для исследования были собраны в 11 метадоновых клиниках в Ляншань-Ийском автономном округе в период с 2004 по 2012 гг. Для анализа связи между ВИЧ-инфекцией и определенными факторами на индивидуальном уровне были использованы модель непространственной логистической регрессии и модель географически взвешенной логистической регрессии.

Результаты: В данном исследовании участвовали 6 458 пациентов. Коэффициент распространенности ВИЧ-инфекции равнялся 25,1%. Модель непространственной регрессии показала положительную связь между нахождением в разводе и ВИЧ-инфекцией. Пространственная модель также показала положительную связь между статусом «в разводе» и ВИЧ-инфекцией, но только среди 49,4% человек, проживающих в некоторых северных округах. Непространственная модель показала отрицательную связь между работой в сфере услуг и ВИЧ-инфекцией. В то же время, пространственная модель показала наличие связи между работой в сфере услуг и ВИЧ-инфекцией, но только среди 23% пациентов, проживающих в некоторых западных округах. Непространственная модель не показала связи между нахождением в браке и ВИЧ-инфекцией в нашей научной дисциплине, но пространственная модель показала отрицательную связь между нахождением в браке и ВИЧ-инфекцией среди 12% человек, проживающих в некоторых западных округах. Для других факторов пространственная и непространственная модели показали схожие результаты.

Заключение: Пространственная модель может быть использована с целью лучшего понимания географической неоднородности в связи между ВИЧ-инфекцией и индивидуальными факторами. Пространственная неоднородность может быть использована с целью адаптации мер по борьбе с ВИЧ-инфекцией для местных регионов, что в свою очередь может привести к более эффективному распределению ограниченных ресурсов для профилактики распространения ВИЧ-инфекции.

Translated from English version into Russian by Irina Zayonchkovskaya, through

Variaciones geográficas en los factores de riesgo asociados con infección por VIH entre consumidores de droga en una prefectura del Sudoeste de China

Yi-Biao Zhou, Qi-Xing Wang , Song Liang, Yu-Han Gong, Mei-Xiao Yang, Yue Chen, Shi-Jiao Nie, Lei Nan, Ai-Hui Yang, Qiang Liao,Yang Yang, Xiu-Xia Song, Qing-Wu Jiang

Resumen

Antecedentes: Estudios previos han mostrado resultados inconsistentes o incluso contradictorios para algunos factores de riesgo asociados con infección por VIH entre consumidores de droga, y éstos pueden explicarse parcialmente debido a variaciones geográficas.

Métodos: Se recolectó información de 11 clínicas de metadona en la Prefectura Autónoma de Liangshan Yi entre los años 2004 y 2012. Se adaptaron un modelo de regresión logística no-espacial y un modelo de regresión logística basado en la geografía para analizar la conexión entre la infección por VIH y factores específicos a nivel individual.

Resultados: En el estudio se registraron 6458 pacientes. La prevalencia de la infección por VIH fue de 25,1%. El modelo no espacial indicó que el estar divorciado estaba positivamente asociado con la infección por VIH. El modelo espacial también mostró que el estar divorciado estaba positivamente asociado con la infección por VIH, pero solo para 49,4% de los individuos que residen en algunos condados del norte. El modelo no espacial sugirió que el trabajo en el sector de servicios estaba negativamente asociado con la infección por VIH. Sin embargo, el modelo espacial indicó que el trabajo en servicios estaba asociado con la infección por VIH, pero solo para 23,0% de los pacientes que viven en algunos de los condados occidentales. El modelo no espacial no mostró que el estar casado estaba asociado con infección por VIH en nuestro estudio de campo, pero el modelo espacial indicó que el estar casado estaba negativamente asociado con la infección por VIH para 12,0% de los individuos que vivían en algunos de los condados occidentales. Para otros factores, el modelo no espacial y el modelo espacial mostraron resultados similares.

Conclusión: El modelo espacial puede ser útil para mejorar la comprensión de la heterogeneidad geográfica en la relación entre infección por VIH y factores individuales. La heterogeneidad espacial puede ser útil para adaptar estrategias de intervención para regiones locales, que pueden consecuentemente resultar en una asignación más eficiente de los escasos recursos para controlar la transmisión del VIH.

Translated from English version into Spanish by Maria Alejandra Aguada, through

TRANSLATORS
WITHOUT BORDERS
